# Metagenomic Analyses Reveal the Involvement of Syntrophic Consortia in Methanol/Electricity Conversion in Microbial Fuel Cells

**DOI:** 10.1371/journal.pone.0098425

**Published:** 2014-05-22

**Authors:** Ayaka Yamamuro, Atsushi Kouzuma, Takashi Abe, Kazuya Watanabe

**Affiliations:** 1 School of Life Sciences, Tokyo University of Pharmacy and Life Sciences, Hachioji, Tokyo, Japan; 2 Graduate School of Science and Technology, Niigata University, Niigata, Niigata, Japan; Graz University of Technology (TU Graz), Austria

## Abstract

Methanol is widely used in industrial processes, and as such, is discharged in large quantities in wastewater. Microbial fuel cells (MFCs) have the potential to recover electric energy from organic pollutants in wastewater; however, the use of MFCs to generate electricity from methanol has not been reported. In the present study, we developed single-chamber MFCs that generated electricity from methanol at the maximum power density of 220 mW m^−2^ (based on the projected area of the anode). In order to reveal how microbes generate electricity from methanol, pyrosequencing of 16S rRNA-gene amplicons and Illumina shotgun sequencing of metagenome were conducted. The pyrosequencing detected in abundance *Dysgonomonas*, *Sporomusa*, and *Desulfovibrio* in the electrolyte and anode and cathode biofilms, while *Geobacter* was detected only in the anode biofilm. Based on known physiological properties of these bacteria, it is considered that *Sporomusa* converts methanol into acetate, which is then utilized by *Geobacter* to generate electricity. This speculation is supported by results of shotgun metagenomics of the anode-biofilm microbes, which reconstructed relevant catabolic pathways in these bacteria. These results suggest that methanol is anaerobically catabolized by syntrophic bacterial consortia with electrodes as electron acceptors.

## Introduction

Methanol is widely used as a precursor in various industrial applications, such as the production of formaldehyde and esters [1], [2], and as a fuel for vehicles and fuel cells [3]. Methanol is also generated as a byproduct in pulp mills and coal gasification plants [4]. Due to its widespread use, methanol is a major pollutant in industrial wastewater, which are often treated in biological treatment plants, such as activated-sludge plants [5]. Although these plants can treat such wastewater effectively, sufficient treatment requires large amounts of electric energy and is therefore of economic and environmental concern.

Microbial fuel cells (MFCs), which exploit living microbes as electrode catalysts, have recently attracted considerable attention as green energy devices for generating electricity from various organic and inorganic materials [6], [7]. In particular, MFCs have the potential to recover energy from biomass wastes and industrial wastewater [8], [9]. In MFCs, microbes degrade pollutants using anodes in place of oxygen as electron acceptors, thereby enabling aeration-free wastewater treatment. In addition, microbes conserve less energy during the generation of electricity, meaning that the amount of sludge discharged during wastewater treatment should be markedly reduced. MFCs are therefore expected to have application in energy- and cost-saving wastewater-treatment processes [10], [11]. In a previous study, Kim and coworkers examined electricity generation in MFCs from methanol and ethanol, but found that MFCs could successfully generate electricity only with ethanol as a fuel source [12]. Thus, it remains to be demonstrated if electricity can be generated from methanol in MFCs.

In the present study, we attempted to generate electricity from methanol in single-chamber MFCs inoculated with activated sludge obtained from an industrial wastewater-treatment plant. Microbial communities that were developed in the MFC were analyzed by pyrosequencing of 16S rRNA-gene amplicons to gain insights into microbes involved in the electricity generation. In addition, the anode metagenome was shotgun-sequenced using an Illumina HiSeq platform for gaining functional and phylogenetic insights into catabolic pathways for the conversion of methanol into electricity.

## Materials and Methods

### Materials

Activated sludge used as an inoculum of MFCs was obtained from a wastewater-treatment facility located within a chemical plant (Gifu, Japan). No specific permissions were required for the sampling. All chemicals used in the present study were reagent grade and purchased from Wako Pure Chemicals (Osaka, Japan) unless otherwise stated. Min ES medium was used as an electrolyte and contained (per liter) 1.2 g K_2_HPO_4_ 0.624 g KH_2_PO_4_ 0.05 g CaCl_2_⋅2H_2_O, 0.165 g MgSO_4_⋅7H_2_O, 0.5 g NH_4_Cl, and 2 ml of a trace elements solution (pH 7.0) [13].

### MFC setup, operation and evaluation

MFC used in the present study was a cylindrical single-chamber reactors (approx. 500 ml in capacity) equipped with a graphite-felt anode (30 cm^2^ in size, 3 mm in thickness) (Sogo Carbon, Kanagawa, Japan) and platinum catalyst-doped membrane-type air cathode (approximately 20 cm^2^ in size and 0.5 mg platinum cm^−2^) that was made as described by Cheng et al. [14]. For operation, the MFC reactor was filled with 500 ml Min ES medium as the electrolyte, which as then bubbled with nitrogen gas treated with a reduced-copper column, and inoculated with activated sludge (approximately 10 g in wet weight). MFCs were operated at 30°C, and the electrolyte was agitated using a magnetic stirrer at approximately 100 rpm. The anode and cathode were connected with an electric wire and *R*_ext_, and the voltage (*E*) across the resistor was measured using a voltage data logger (HA-1510; Graphtec, Kanagawa, Japan). Current (*I*, in amperes [A]) was calculated using the equation *I* =  *E*/*R* (where *V* is the cell voltage in volts [V] and *R* is the resistance in ohms [Ω]), and current density (*J*; mA m^−2^) and power density (*P*; mW m^−2^) were calculated using the projected area of anode. Coulombic efficiency (*ε*_c_) was calculated by dividing the total coulombs transferred to the anode by the theoretical maximum number of coulombs (total coulombs produced by complete methanol oxidation to carbon dioxide), as described elsewhere [6]. Polarization curves were generated using a potentiostat (HSV-100; Hokuto Denko, Tokyo, Japan), from which open-circuit voltage (*V*_oc_), short-circuit current density (*I*_sc_), and maximum power density (*P*_max_) were estimated as described elsewhere [6].

In an anode-exchange experiment, a spent anode was transferred from a MFC operating at a cell voltage of approximately 0.3 V to a MFC reactor containing fresh electrolyte (10 mM methanol), and cell voltage was immediately monitored. A fresh anode was also inserted into the original MFC reactor, and cell voltage was monitored after supplementation of the electrolyte with 10 mM methanol.

### Chemical analyses

Methanol was measured using a gas chromatograph (GC-2014; Shimadzu, Kyoto, Japan) equipped with a flame ionization detector (Shimadzu) and Stabilwax column (length, 30 m; inner diameter, 0.32 mm; film thickness, 0.25 µm; Restek, Bellefonte, PA). Operation temperatures were as follows: injection temperature, 250°C; column temperature, 40 to 240°C (increased at a rate of 10°C min^−1^); and detector temperature, 300°C. Organic acids, including, formate, malate, lactate, acetate, citrate, succinate, maleate, propionate, and fumarate, in electrolytes were measured using high-performance liquid chromatography (HPLC; Agilent Technologies, Tokyo, Japan) after the cells were removed by filtration through a cassette membrane (0.22-µm pore size, DISMIC-13HP; Advantec, Tokyo, Japan) as described elsewhere [15]. Methane, hydrogen, nitrogen, and carbon dioxide concentrations in the MFC headspace were measured using a gas chromatograph (GC-2014; Shimadzu) equipped with a thermal conductivity detector and molecular sieve 5A 60–80/Porapack Q 80–100 column (Shimadzu) as described elsewhere [16]. The column, injection, and detector temperatures were 50, 100, and 80°C, respectively.

### Protein content

To determine total protein content, pieces (0.3 cm^2^) of the graphite-felt anode and air cathode (0.3 cm^2^) were placed in a tube containing B-PER II Bacterial Protein Extraction Reagent (Pierce, Rockford, IL, USA) to solubilize proteins according to the manufacturer's instructions. Planktonic cells in the electrolyte (1 ml) was collected by centrifugation at 5,000×*g* for 10 min and then re-suspended in B-PER II reagent. Protein concentrations in these suspensions were determined using the Micro BCA Protein Assay Kit (Pierce) according to the manufacturer's instructions. Total protein content was calculated based on the anode-projection area, cathode area, or volume of the electrolyte.

### Pyrosequencing of 16S rRNA gene amplicons

DNA was extracted from biofilms formed on the graphite-felt anode (1 cm^2^) and cathode membrane (1 cm^2^), and from planktonic cells in the electrolyte (1 ml) using the FAST DNA Spin Kit for Soil (Q-Bio, Carlsbad, CA, USA). PCR amplification of 16S rRNA gene fragments (V1–V3 region) was performed using primers ad-tag-8F and ad-533R, in which the underlined sequences were adaptors added for pyrosequencing and XXXXXX was an arbitrary tag sequence for sample identification [17]. PCR conditions were described elsewhere [17]. Amplicons were purified using a QIAquick PCR Purification Kit (Qiagen, Tokyo, Japan). Amplicons from different samples were mixed at the same concentration (1 ng µl^−1^ each), and were then subjected to pyrosequencing using a Genome Sequencer FLX system at the Dragon Genomics Center (Mie, Japan). Phylogenetic analyses were conducted using the DDBJ 16S rRNA database (released on Feb. 21, 2012) and the RDP classifier [18]. An operational taxonomic unit (OTU) was defined as a unique sequence or group of sequences with sequence homologies of over 97%. Alignment of sequences and construction of trees were conducted using the MEGA program ver. 5.1[19].

### Shotgun metagenomics

Total genomic DNAs were extracted from pieces of graphite-felt anode (3 cm2 in total area) using the FastDNA SPIN kit for soil. DNA quality was assessed by agarose-gel electrophoresis, spectophotometeric analysis, and Quant-iT dsDNA BR assay kit (Invitrogen, Carlsbad, CA). Approximately 5 µg of quality-checked DNA was used for library construction and subsequent sequence analyses. High-throughput sequencing of the metagenome samples was performed in the Dragon Genomics Center (Mie, Japan) using a quarter of a lane in the HiSeq 2000 sequencing system (Illumina, San Diego, CA) as described elsewhere [20]. Contig assembly gene prediction, and annotation were conducted as described previously [20]. BLASTP in the BLAST+ software was used to align translated protein sequences to the NCBI-NR database (released in February 2013) with an e-value of 10^−2^ [21]. The resulting BLASTP files were analyzed according to NCBI taxonomy using MEGAN software version 4.70.4 with the following LCA parameters: Min score, 50; top percent, 10.0; Min support, 5 [22]. The KEGGviewer module in MEGAN was used to map the sequences in each sample to KEGG pathways [23]. Program PEMS, which is based on Tetranucleotide BLSOM was used for the taxonomic assignment of metagenome contigs [24].

Nucleotide sequences determined in the present study have been deposited into the DDBJ Sequence Read Archive database (Accession Number: DRA001173). In addition, metagenome sequences will be provided on request.

## Results and Discussion

### Electricity generation from methanol

Single-chamber MFCs were inoculated with activated sludge and fed methanol as the sole source of carbon and energy. *E* was monitored during the operation, and the MFCs were re-supplemented with methanol when *E* fell to 0.05 V (a typical *ET* curve is shown in Fig. S1). *R*_ext_ was initially 10,000 Ω, but rapidly dropped to 1,000 Ω, and finally stabilized at 510 Ω. As shown in Fig. S1, *E* immediately increased in response to supplementation of the MFC with methanol. Methane was initially detected in the MFC headspace, but the partial pressure in the head space was decreased down to an undetectable level after 60 days of operation (data not shown).

**Figure 1:**
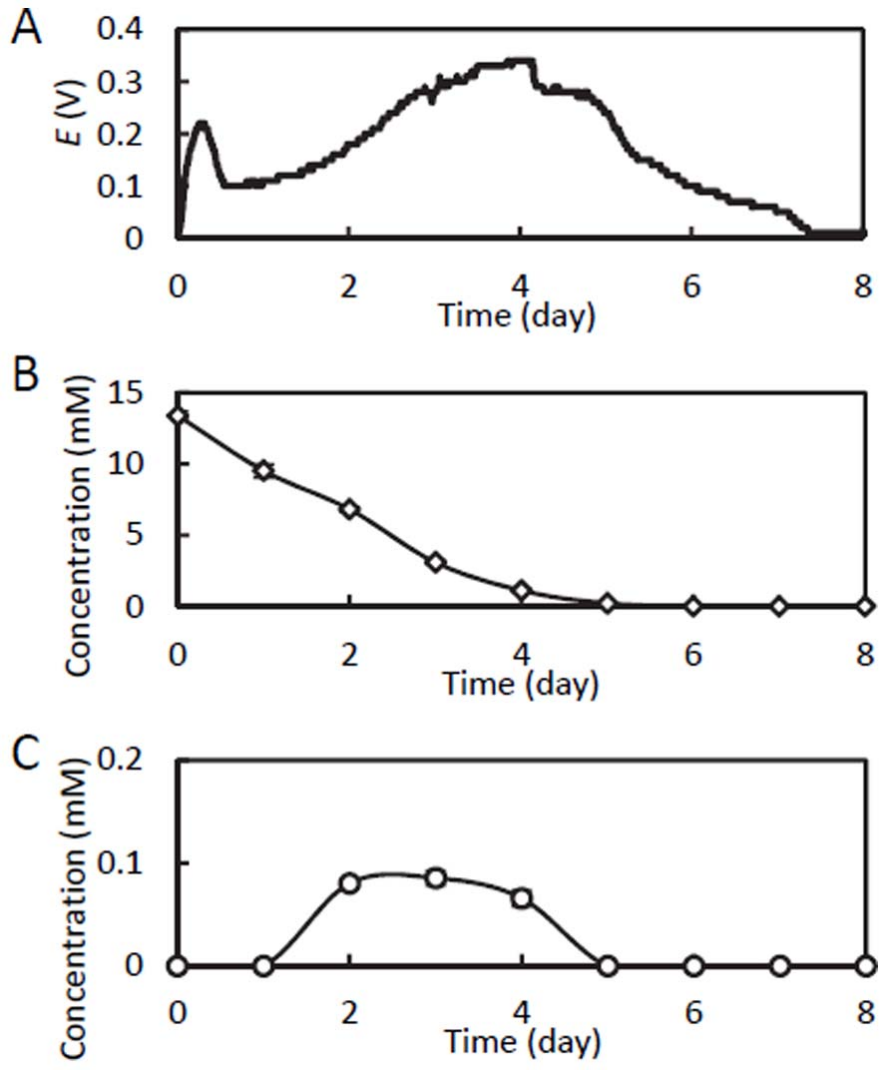
Typical time courses of cell voltage (A), methanol concentration (B), and acetate concentration (C), after supplementation of the MFC with 10 mM methanol. In panels B and C, data are means ± SD (n = 3), and error bars are shown when they are larger than symbols.

**Figure 2:**
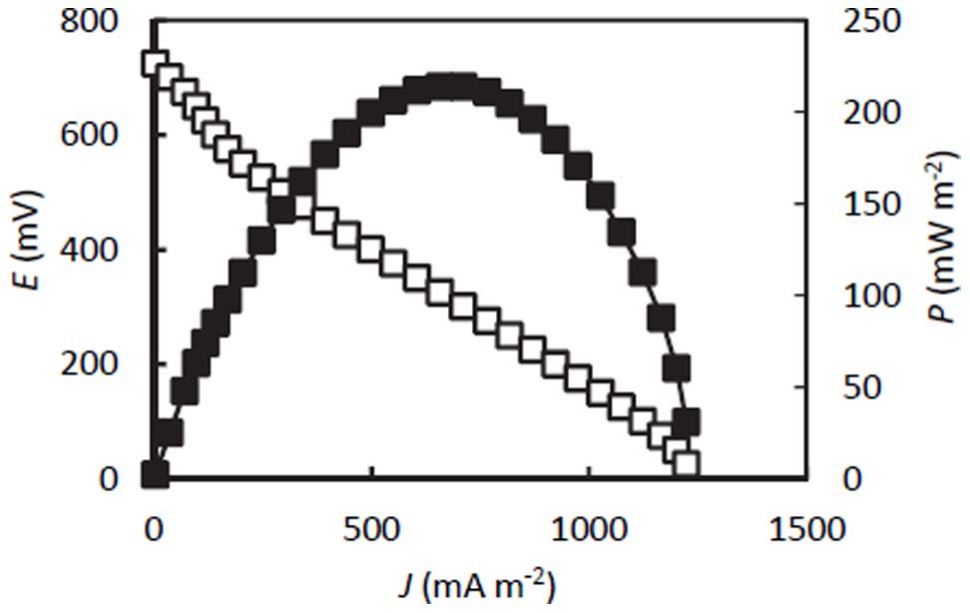
Polarization (open squares) and power (closed squares) curves for the methanol-fed MFC.

A typical *E* curve after supplementation of the MFC with 10 mM methanol is shown in Fig. 1A. During MFC operation under *R*_ext_ of 510Ω, Coulombic efficiency (*ε*_c_) was estimated to be 13±3.3% (mean ± SD, n = 3). When *E* increased to over 0.3 V, polarization analysis was conducted for the methanol-fed MFC. Using the obtained polarization and power curves (Fig. 2), *V*_oc_, *P*_max_ and *J*_sc_ were determined to be 720 mV, 220 mW m^−2^, and 1200 mA m^−2^, respectively.

During operation of the methanol-fed MFC, the concentrations of methanol and metabolites were analyzed by GC and HPLC (Fig. 1B). These analyses revealed that methanol relatively rapidly disappeared, and that electricity generation continued even after the depletion of methanol. Among the organic acids examined by HPLC, only acetate was detected, albeit temporarily, reaching a concentration of 0.1 mM (Fig. 1C). Methane and hydrogen were not detected in the GC analysis, suggesting that these compounds were not intermediate metabolites. From these data, it was suggested that acetate was an intermediate metabolite produced from methanol in microbial catabolic processes for electricity generation.

### Distribution of microbes in the methanol-fed MFC

**Figure 3:**
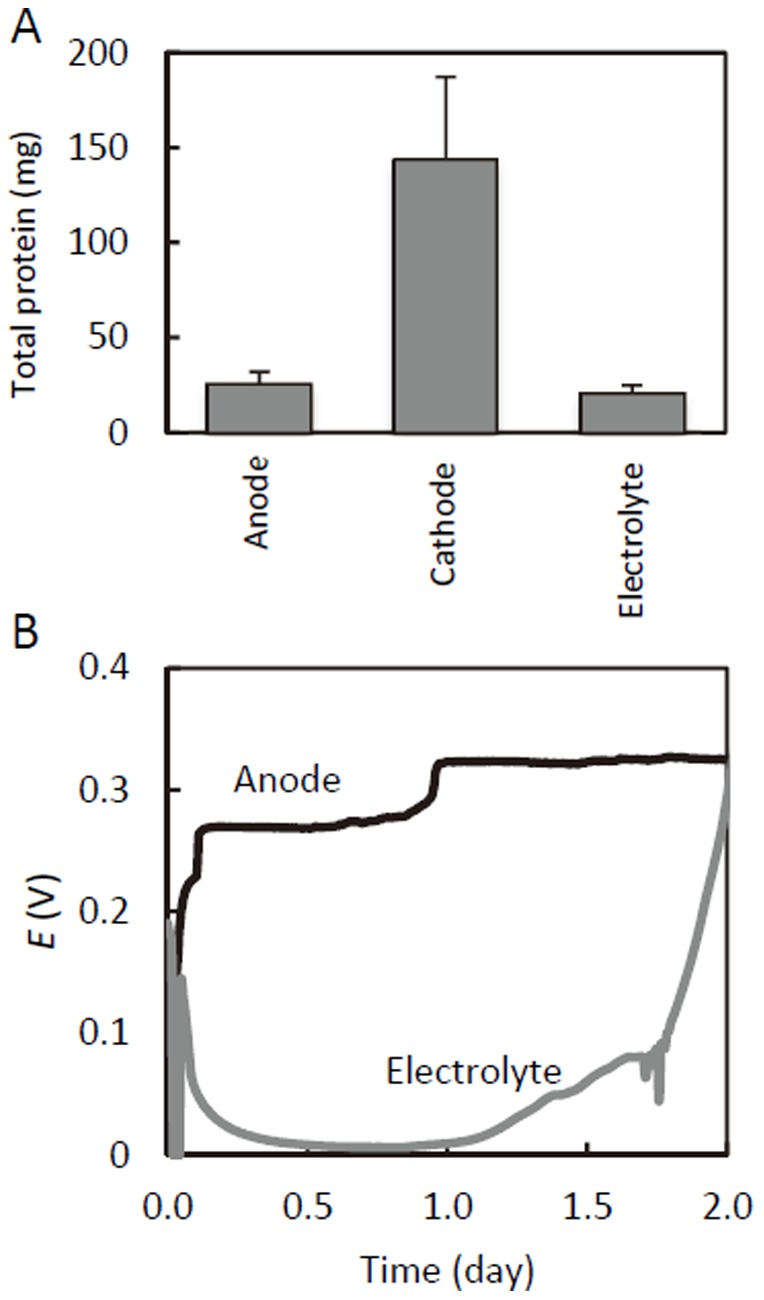
Distribution of microbes in the methanol-fed MFC. (A) Total protein contents showing amounts of microbes associated with the anode biofilm, cathode biofilm, and electrolyte. (B) Results of an anode-exchange experiment, in which cell voltages of methanol-fed MFCs were measured after the microbe-bearing anode was transferred to a reactor containing fresh electrolyte (black line) and a new anode was placed in the spent electrolyte of the initial reactor (gray line).

After electricity was stably generated in the methanol-fed MFC, microbes were observed attached to the graphite-felt anode, and the electrolyte was slightly turbid, suggesting the presence of planktonic microbes. In addition, a thick biofilm adhering to the inner surface of the air cathode (directly exposed to the electrolyte) was also visible. To compare the amount of microbes present in each of the three parts (anode, cathode, and electrolyte) of the MFC reactor, the total protein content was determined (Fig. 3A). The analysis showed that the cathode biofilm had approximately five-fold more proteins than the anode biofilm and electrolyte. It is likely that cathode-biofilm microbes respired oxygen that penetrated though the air cathode, resulting in a relatively large amount of methanol being consumed to support their growth. The proliferation of cathode biofilm would have been the major cause for the lowered *ε*_c_ in the methanol-fed MFC. The growth of planktonic microbes in the MFC was also observed; it is conceivable that sulfate in the electrolyte served as an electron acceptor for these microbes.

Previous studies have suggested that microbes are able to transfer electrons to anodes through direct contact with anode surfaces or via water-soluble mediators [25]. To evaluate and compare the contribution of anode-biofilm and planktonic microbes to electricity generation in the methanol-fed MFC, an anode-exchange experiment was conducted, in which the anode from an MFC operated for 205 days was transferred to a new MFC reactor with the fresh electrolyte (Fig. 3B). The cell voltage of MFC equipped with the biofilm-bearing spent anode (anode MFC) increased to over 0.2 V within several hours of operation. For the original MFC containing planktonic microbes in the spent electrolyte and a fresh graphite-felt anode (electrolyte MFC), the cell voltage was nearly 0.2 V immediately after supplementation with methanol at the start of operation, dropped to 0.01 V within several hours, and began increasing again after one day of operation. The initial cell voltage of the electrolyte MFC is considered to have been generated abiotically from reducing equivalents accumulated in the electrolyte, and not by microbial catabolic activities. The data therefore suggest that the electricity in the methanol-fed MFC was predominantly generated by anode-biofilm microbes.

### Molecular phylogenetic analyses of bacteria in the methanol-fed MFC

**Figure 4:**
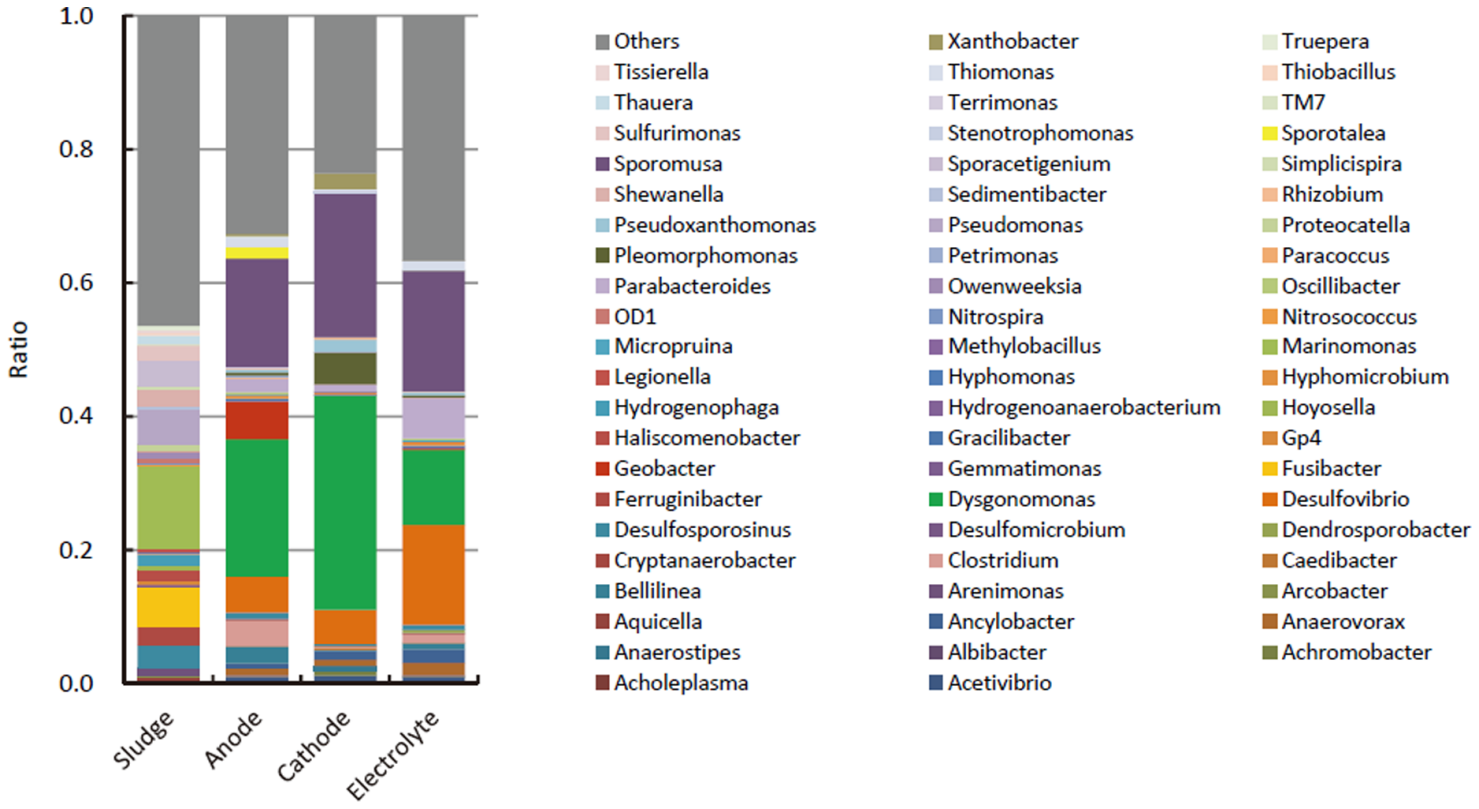
Relative abundances of genus-level phylogenetic groups based on pyrosequenced 16S rRNA-gene amplicons showing structures of bacterial communities in the original sludge, anode biofilm, cathode biofilm, and electrolyte.

To gain phylogenetic information about the microbial species occurring in the methanol-fed MFC, pyrosequencing of 16S rRNA gene amplicons was conducted. We compared the bacterial composition of the anode and cathode biofilms, and electrolyte to identify differences that could be associated with variations in electron acceptors (Fig. 4 and Table S1). We found that the bacterial composition of the MFC samples largely differed from that in the original activated sludge. The bacterial genus most abundantly detected in the MFC samples was *Dysgonomonas*, followed by *Sporomusa* and *Desulfovibrio*. *Dysgonomonas* was particularly abundant in the cathode biofilm, whereas *Desulfovibrio* had increased most in the electrolyte. In addition, several genera were present specifically in one region of the MFC, including *Geobacter* in the anode biofilm, and *Pleomorphomonas*, *Pseudoxanthomonas*, and *Xanthobacter* in the cathode biofilm. Physiological characteristics relevant to the occurrence of these genera are discussed below.

*Dysgonomonas* is a member of the phylum *Bacteroidetes* and was first described for facultatively anaerobic clinical isolates [26]. Recently, sequence types affiliated with this genus were detected abundantly in MFCs treating model organic waste, consisting of starch, peptone, fish extracts [27], [28]. In addition, *D. oryzarvi* strain Dy73 was isolated from an MFC, although this strain was unable to generate electricity in pure culture [29]. It is currently unclear how *Dysgonomonas* persists in MFCs. In addition, the metabolism of methanol by *Dysgonomonas* has not been reported. In our analysis, sequences affiliated with *Dysgonomonas* were categorized into two major OTUs (Figs. S2A and S2B), with OTU C251, which is closely related to *D. oryzarvi*, being more abundantly detected than OTU E279. It is interesting that similar *Dysgonomonas* sequences to those found here were also detected in MFCs treating high molecular-weight organics [27], [28]. Although the physiological roles of *Dysgonomonas* in MFCs still remain enigmatic, metagenomic analyses provide some insights into their metabolism (see below).

The second most abundant genus in the methanol-fed MFC was *Sporomusa* (Fig. 4), a member of the phylum *Firmicutes* [30]. Conspicuous metabolic features that are common among strains of this genus include homoacetogenesis, utilization of C1 compounds, including methanol and decarboxylation of dicarboxylic acids [30]. *Sporomusa* is known to oxidize methanol to form acetate according to the following equation [31]:
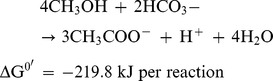
In addition, syntrophic associations of *Sporomusa* with methanogens and sulfate-reducing bacteria allow methanol oxidation to be coupled to methanopgenesis and sulfate reduction, respectively, via interspecies hydrogen transfer [31]–[33]. Prior to the present study, however, this genus had not been identified as a major component in MFCs, probably because methanol-fed MFCs have not been developed previously. Our phylogenetic analysis detected three major OTUs (A74, A180, and E305) that were related to the genus *Sporomusa* (Figs. S2C and S2D), although two did not appear to be authentic *Sporomusa*. OTU A180, which was the most abundant *Sporomusa* OTU, is closely related to *S. sphaeroides,* able to oxidize C1 compounds [30]. Because our metabolite analyses (Fig. 1C) detected acetate in the course of methanol degradation, it is likely that *Sporomusa* converted methanol to acetate in the methanol-fed MFC.

*Desulfovibrio* was also detected abundantly in the methanol-fed MFC, particularly in the electrolyte (Fig. 4). Analyses of *Desulfovibrio* sequences identified several major OTUs (Figs. S2E and S2F), among which OTU A41 was the most abundant. Two possible niches for *Desulfovibrio* may exist in the methanol-fed MFC. First, as a number of *Desulfovibrio* strains are capable of utilizing alcohols, including methanol, as carbon and energy sources [34], it is possible that methanol was oxidized by these bacteria using sulfate as the electron acceptor. Second, *Desulfovibrio* has been shown to form syntrophic interactions with *Sporomusa* to facilitate methanol oxidation coupled to sulfate reduction [32]. In previous studies, *Desulfovibrio* has also been detected as major constituents in MFCs, and it has been suggested that sulfate may serve as an electron shuttle between sulfate-reducing bacteria (such as *Desulfovibrio*) and the anode [35]. In the case of the methanol-fed MFC, however, since the sulfate concentration in the electrolyte was low (approximately 0.67 mM), we consider that *Desulfovibrio* did not largely contribute to electricity generation. This idea is also supported by the finding that *Desulfovibrio* was abundant in the planktonic community in the electrolyte that only partially contributed to electricity generation.

The genus *Geobacter* has frequently been detected in various types of MFCs, and it thought to couple the oxidation of acetate, hydrogen and/or formate to generation of electricity [36]. In the methanol-fed MFC, *Geobacter* represented a substantial portion of the bacterial community only in the anode biofilm (Fig. 4), suggesting that they grew via anode respiration. Most of the *Geobacter* sequences (over 95%) detected in the anode biofilm were closely related to *G. sulfurreducens* (Figs. S2G and S2G), which has been intensively studied for its ability to transfer electrons to extracellular electron acceptors [36]. Because the metabolite analysis detected acetate in the electrolyte of the methanol-fed MFC, it is likely that *Geobacter* cells grew by oxidizing acetate and using the anode as an electron acceptor.

In the cathode biofilm, several genera, including *Pleomorphomonas*, *Pseudoxanthomonas*, and *Xanthobacter*, were specifically detected (Fig. 4, and Table S1). *Pleomorphomonas* and *Xanthobacter* include strains that are able to utilize methanol under aerobic conditions [37], [38]. Concerning the genus *Pseudoxanthomonas*, although no strains have been demonstrated to grow on methanol, a genomic analysis has shown that *P. spadix* encodes methanol dehydrogenase [39]. We suggest that members of these bacterial genera grew on methanol with oxygen that had diffused through the air cathode as the electron acceptor in the cathode biofilm.

### Metagenomic analyses of anode-biofilm microbes

The aforementioned phylogenetic analyses suggest the possibility that electricity was generated, at least partly, in the methanol-fed MFC through a syntrophic association between *Sporomusa* and *Geobacter*, in which *Sporomusa* converted methanol into acetate, and it was then oxidized by *Geobacter* to generate electricity. To evaluate this hypothesis and gain insight into catabolic pathways involved in the conversion of methanol into electricity, we performed shotgun sequencing of metagenome samples extracted from the anode biofilm in the methanol-fed MFC. Approximately 55 mega reads (5.5 gigabase) were generated using the Illumina platform and applied to the contig assembly. Numerical data for the sequencing and assembly are summarized in Table 1.

**Table 1:** Summary of numerical data for the metagenomic analyses of microbes associated with the anode biofilm in the methanol-fed MFC.

Item	Value
Total read length (bp)	5,511,432,200
No. of contigs	78,882
Average contig length (bp)	1,348
Total contig length (bp)	106,321,402
No. of large contigs (>50 kb)	278
Total length of large contigs (bp)	30,256,000
No. of genes predicted in large contigs	16054

**Figure 5:**
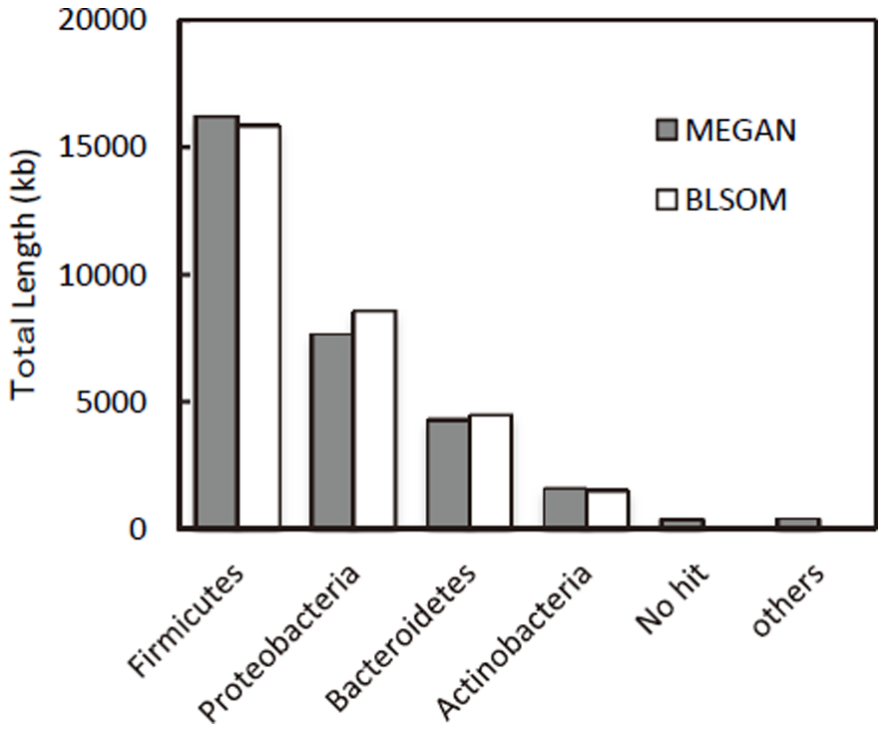
Comparison of the total lengths of large contigs affiliated with different phyla as determined by MEGAN or BLSOM analyses of the metagenome data.

In the shotgun metagenome approach, large contigs with high read coverages must represent genomes of abundantly occurring microbes. In the present study, we concentrated our subsequent analyses on large contigs (>50 kb) that represented approximately 30% of the total contig length (Table 1). These contigs were subjected to MEGAN and BLSOM analyses for phylogenetic assignments at the phylum level (Fig. 5 and Table S2), and the functions of detected genes were predicted by BLAST analyses (Table S2). From the comparisons of the total lengths of large contigs affiliated with the different phyla (Fig. 5), it is shown that the MEGAN and BLSOM results agreed well with each other, and that the total contig length affiliated with the *Firmicutes* was the largest (approx. 16 Mb), followed by those for the *Proteobacteria* (approx. 8 Mb) and *Bacteroidetes* (approx. 5 Mb). This finding is consistent with the molecular phylogenetic analyses based on 16S rRNA gene sequences (Fig. 4), in which members of these three phyla were abundantly detected.

We attempted to reconstruct the catabolic pathways involved in the conversion of methanol into electricity. The MEGAN program was used to assign predicted genes to KEGG metabolic maps (Fig. S3), and catabolic genes were phylogenetically assigned based on taxa identified at the contig level (Table S2). In the KEGG map, methanol catabolism proceeds by two pathways, involving either one involving methanol dehydrogenase or methanol:coenzyme M (CoM) methyltransferase (Fig. S3A). To date, methanol dehydrogenases have mainly been found in aerobic methylotrophs [40], whereas methanol:coenzyme M (CoM) methyltransferases are found in methanogenic archaea [41]. In our analyses, neither pathway was completed with the genes predicted in the metagenome contigs, suggesting that an alternative pathway was necessary for the catabolism of methanol.

**Figure 6:**
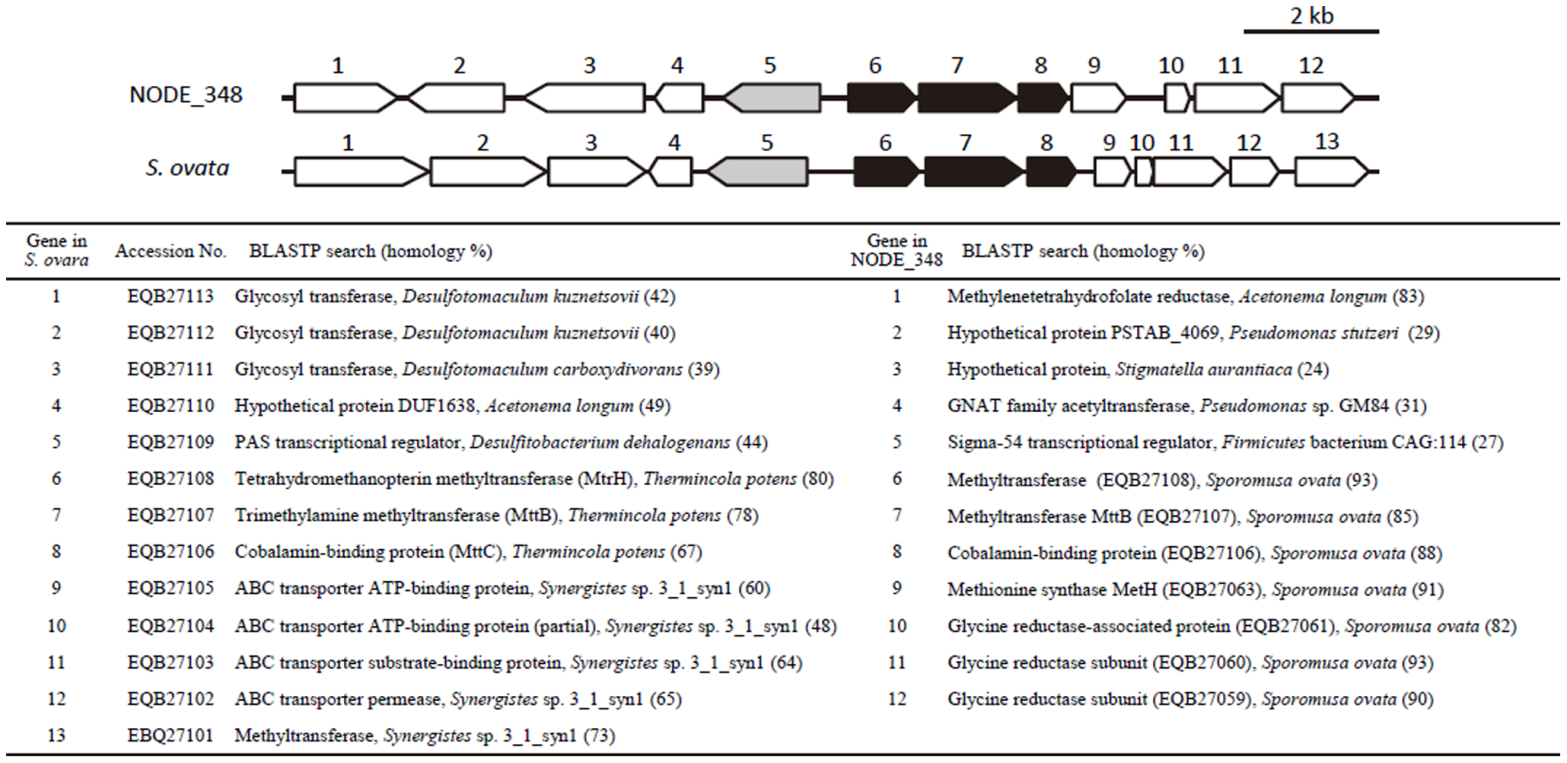
Gene clusters containing putative methanol:THF methyltransferases (black arrows) in metagenome contig NODE_348 and the genome of *Sporomusa ovata*. Possible genes encoding transcriptional regulators for methyltransferases are indicated with gray arrows. Results of BLAST search for the genes are described in the table.

*Sporomusa* is known to convert methanol into acetate under anaerobic conditions [31] using corrinoid-dependent methyltransferases [42]. In this organism, however, methyl group is transferred to tetrahydrofolate (THF) rather than CoM [42]. In the present metagenomic analyses, putative genes homologous to *mttB* and *mttC*, which encode two subunits of trimethylamine:CoM methyltransferase, were detected (Fig. S3A), but no homologues of the third subunit (MttA) were found. MttA, MttB and MttC are homologues of MtaA, MtaB and MtaC, respectively, of methanol:CoM methyltransferase; in these enzymes, subunit B is suggested to activate the methyl group in a substrate (e.g., methanol and trimethylamine) and transfer it to cobalamin bound to subunit C, and subunit A then transfers the methyl group to CoM [41]. Here, we investigated genetic organization of regions peripheral to the identified *mttB* and *mttC* homologues and found that genes that exhibit homology to *mtrH* are associated with them (Fig. 6). MtrH is a subunit of methyltetrahydromethanopterin:CoM methyltransferase in methanogens and catalyzes a methyl-transfer reaction between cobalamin and tetrahydromethanopterin, a derivative of THF [43]. A similar gene cluster is also found in the genome of *Sporomusa ovata* (Fig. 6) [44]. The presence of the MtrH-like subunit in association with MttBC suggests that this gene cluster encodes a methanol:THF methyltransferase, which is consistent with the above-described physiological study [42]. A number of genes in contig NODE_348 are highly homologous to those in *S. ovata*, supporting the idea that members of the genus *Sporomusa* were involved in the initial step in methanol catabolism in the methanol-fed MFC.

**Figure 7:**
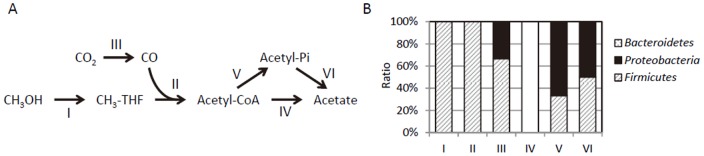
Catabolic pathway for methanol/acetate conversion in the methanol-fed MFC predicted from the metagenome data (A), and phylum-level distributions of genes assigned to each catabolic step (B). Step I, methanol:THF methyltransferase; II, acetyl-CoA synthase (EC.2.3.1.169); III, carbon monoxide dehydrogenase (EC.1.2.7.4); IV, acetyl-CoA synthetase (EC.6.2.1.1); V, phosphate acetyltransferase (EC.2.3.1.8); and VI, acetate kinase (EC.2.7.2.1).

Acetate can be synthesized from methylated THF by using a part of the Wood-Ljungdahl pathway for acetogenic carbon fixation in prokaryotes [45], and genes for enzymes in this part were found in metagenome contigs (Fig. S3B). Based on the genes identified in the metagenome sequencing analysis, we proposed a catabolic pathway for the conversion of methanol to acetate, and determined the phylogenetic affiliation of genes involved in each step (Fig. 7). The analysis revealed that the entire pathway (methanol to acetate) is possible with genes in contigs affiliated with the *Firmicutes*. The initial two steps can be catalyzed only by members of the *Firmicutes*, whereas the conversion of acetyl-coenzyme A (CoA) to acetate (steps IV, V and VI) can also be performed by members of other phyla. The results suggest that *Sporomusa* is involved in the conversion of methanol into acetate in the methanol-fed MFC.

The initial step in acetate catabolism is the activation of acetate to produce acetyl-CoA, and this can be accomplished through two pathways (Figs. 7, and 3B). The metagenomic data showed that acetate can be catalyzed by members of the *Firmucutes*, *Proteobacteria*, and *Bacteroidetes* (Fig. 7), whereas genes for acetyl-CoA synthase were found only in contigs affiliated with the *Bacteroidetes* (Fig. 7). It is likely that members of the *Bacteroidetes*, including *Dysgonomonas*, utilize acetyl-CoA synthase for growth on acetate. A previous study has shown that *G. sulfurreducens* utilizes acetate kinase and phosphotransacetylase for the acetate-activation reaction [46]; here, genes for these enzymes were found in metagenome contigs affiliated with the *Deltaproteobacteria* (e.g., NODE_12 and NODE_122, see Table S2). In a downstream pathway, acetyl-CoA may be incorporated into the citrate cycle, and genes encoding enzymes in this cycle are found in metagenome contigs (Fig. S3C and Table S2). In contigs affiliated with the *Deltaproteobacteria*, most genes for the citrate-cycle enzymes were found along with those encoding cytochromes, such as PpcA and OmcE, involved in the extracellular electron-transport pathway of *Geobacter* (Table S2). These cytochromes are widely distributed among *Geobacter* species [47]. Genes for some other multiheme cytochromes (MHC) were also found in contigs affiliated with the *Deltaproteobacteria* (Table S2), and were highly homologous to genes in *Geobacter*. Together, these data support the possibility that electricity was generated from acetate by members of the genus *Geobacter*.

### Conclusions

The present study developed MFCs generating electricity from methanol, and analyzed microbes and catabolic pathways involved in the methanol/electricity conversion. Results suggest that the syntrophic consortium comprised of *Sporomusa* and *Geobacter* contributes, at least partly, to the conversion of methanol into electricity. These bacteria have also been detected abundantly in continuous-flow MFC treating methanol-containing artificial wastewater (unpublished data), suggesting that the *Sporomusa*/*Geobacter* interaction is ubiquitous in the methanol/electricity conversion. Few studies have demonstrated the electricity generation from C1 compounds [48], and, to our knowledge, this is the first report describing catabolic pathways for C1 compounds that are associated with electricity generation. In future studies, we will conduct metatranscriptomic analyses to understand the regulation of enzymes in the relevant catabolic pathways. Direct PCR cloning of catabolic genes from metagenomes may also be possible.

## Supporting Information

Figure S1 **Time course of cell voltage (** ***E*** **) during the enrichment of microbial communities generating electricity from methanol in the single-chamber MFC.**
(TIF)

Figure S2 **Neighbor-joining trees based on 16S rRNA-gene sequences showing phylogenetic positions of major sequence types related to the genera** ***Dysgonomonas*** **(A),** ***Sporomusa*** **(C),** ***Desulfovibrio*** **(E) and** ***Geobacter*** **(G).** Relative abundances of major sequence types related to *Dysgonomonas* (B), *Sporomusa* (D), *Desulfovibrio* (F) and *Geobacter* (H) are also shown.
(TIF)

Figure S3 **KEGG-pathway maps for methane metabolism (A), carbon fixation in prokaryotes (B) and citrate cycle (C).** Genes found in the metagenome contigs were highlighted in green (color strength corresponds to gene abundance in the metagenome).
(PDF)

Table S1 (DOCX)

Table S2 (DOCX)
