# Supplementary figures and images for: Metagenomic Analyses Reveal the Involvement of Syntrophic Consortia in Methanol/Electricity Conversion in Microbial Fuel Cells
Source: PLoS One. 2014 May 22;9(5):e98425. doi: 10.1371/journal.pone.0098425 (PMC4031174; doi:10.1371/journal.pone.0098425)

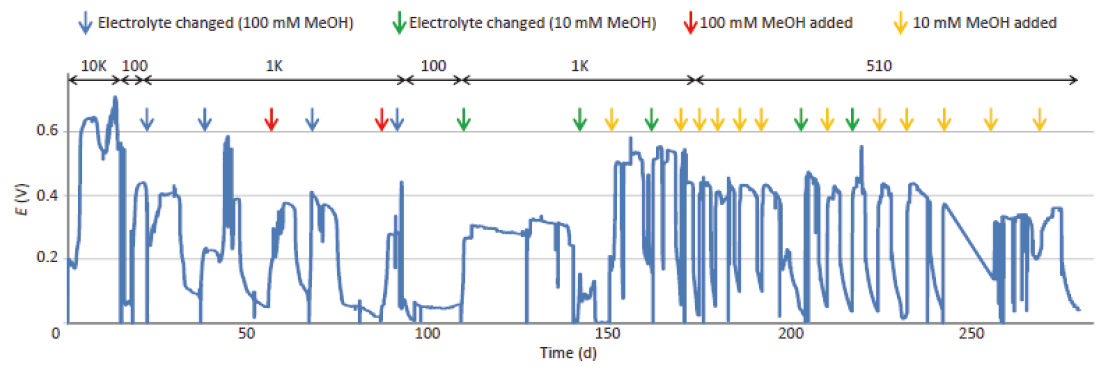

Supplement: Figure S1 — Time course of cell voltage ( E ) during the enrichment of microbial communities generating electricity from methanol in the single-chamber MFC. (TIF) [file pone.0098425.s001.tif]

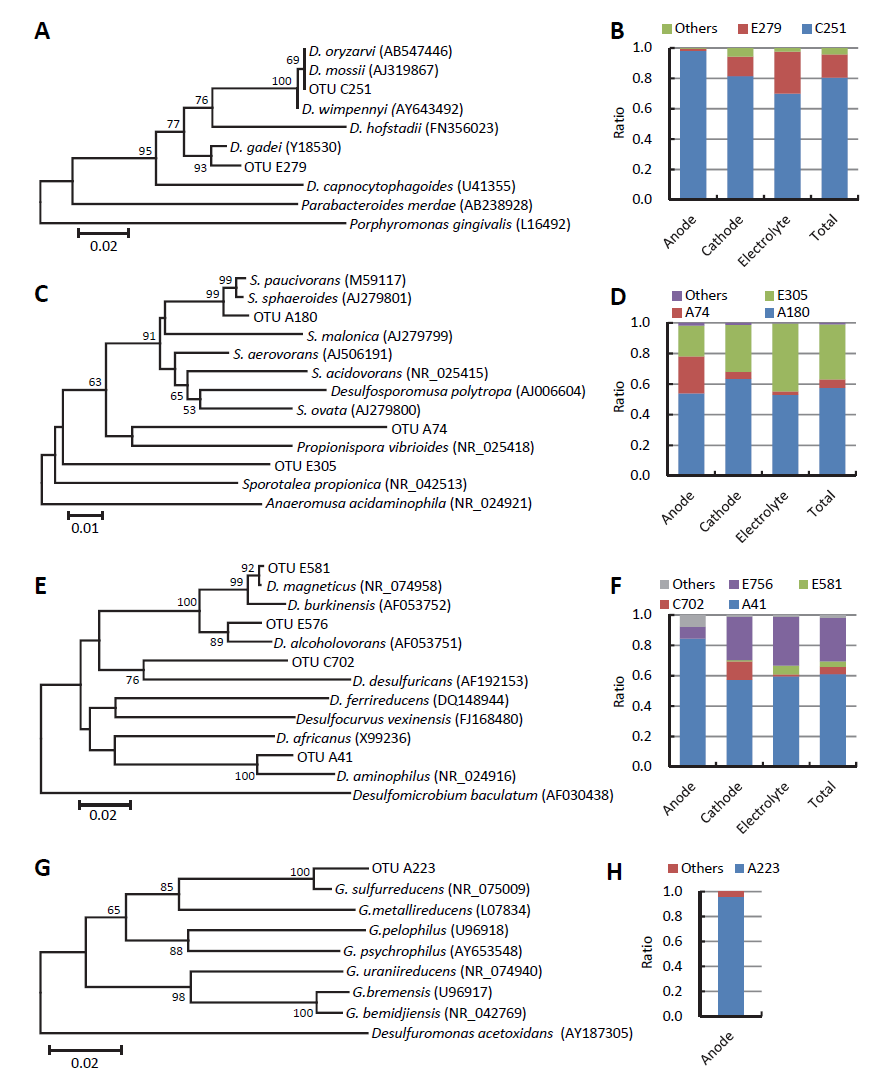

Supplement: Figure S2 — Neighbor-joining trees based on 16S rRNA-gene sequences showing phylogenetic positions of major sequence types related to the genera Dysgonomonas (A), Sporomusa (C), Desulfovibrio (E) and Geobacter (G). Relative abundances of major sequence types related to Dysgonomonas (B), Sporomusa (D), Desulfovibrio (F) and Geobacter (H) are also shown. (TIF) [file pone.0098425.s002.tif]

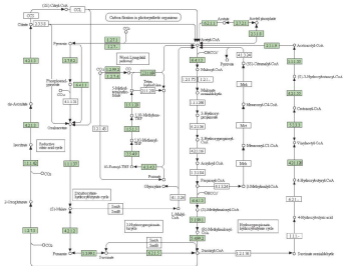

## CITRATE CYCLE CTGA CYCLES

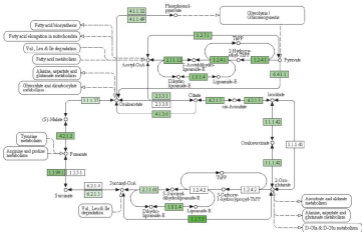

Supplement: Figure S3 — KEGG-pathway maps for methane metabolism (A), carbon fixation in prokaryotes (B) and citrate cycle (C). Genes found in the metagenome contigs were highlighted in green (color strength corresponds to gene abundance in the metagenome). (PDF) [file pone.0098425.s003.pdf]
