# Supplementary material for: Metagenomic Analyses Reveal the Involvement of Syntrophic Consortia in Methanol/Electricity Conversion in Microbial Fuel Cells
Source: PLoS One. 2014 May 22;9(5):e98425. doi: 10.1371/journal.pone.0098425 (PMC4031174; doi:10.1371/journal.pone.0098425)
Supplement: Table S1 — (DOCX) [file pone.0098425.s004.docx]

**Table S1** Major genera (n > 10) detected in the pyrotag sequencing of 16S rRNA-gene amplicons for the original activated sludge, anode and cathode biofilms and planktonic cells in the electrolyte.

| Taxonomic group | Activated sludge | | Anode biofilm | | Cathode biofilm | | Electrolyte | |
| --- | --- | --- | --- | --- | --- | --- | --- | --- |
|  | No. | % | No. | % | No. | % | No. | % |
| *Acetivibrio* | 0 | 0.0 | 22 | 1.0 | 96 | 1.2 | 87 | 1.0 |
| *Acholeplasma* | 25 | 0.5 | 0 | 0.0 | 0 | 0.0 | 0 | 0.0 |
| *Achromobacter* | 0 | 0.0 | 1 | 0.0 | 52 | 0.7 | 4 | 0.0 |
| *Albibacter* | 0 | 0.0 | 5 | 0.2 | 1 | 0.0 | 16 | 0.2 |
| *Anaerostipes* | 0 | 0.0 | 0 | 0.0 | 64 | 0.8 | 0 | 0.0 |
| *Anaerovorax* | 7 | 0.1 | 24 | 1.1 | 75 | 0.9 | 163 | 1.9 |
| *Ancylobacter* | 0 | 0.0 | 17 | 0.8 | 104 | 1.3 | 173 | 2.0 |
| *Aquicella* | 16 | 0.3 | 0 | 0.0 | 0 | 0.0 | 0 | 0.0 |
| *Arcobacter* | 18 | 0.3 | 0 | 0.0 | 0 | 0.0 | 0 | 0.0 |
| *Arenimonas* | 53 | 1.0 | 0 | 0.0 | 0 | 0.0 | 0 | 0.0 |
| *Bellilinea* | 0 | 0.0 | 59 | 2.6 | 11 | 0.1 | 81 | 0.9 |
| *Caedibacter* | 0 | 0.0 | 2 | 0.1 | 26 | 0.3 | 7 | 0.1 |
| *Clostridium* | 0 | 0.0 | 85 | 3.8 | 15 | 0.2 | 104 | 1.2 |
| *Cryptanaerobacter* | 0 | 0.0 | 6 | 0.3 | 8 | 0.1 | 18 | 0.2 |
| *Dendrosporobacter* | 0 | 0.0 | 0 | 0.0 | 4 | 0.1 | 40 | 0.5 |
| *Desulfomicrobium* | 0 | 0.0 | 2 | 0.1 | 0 | 0.0 | 4 | 0.0 |
| *Desulfosporosinus* | 179 | 3.5 | 20 | 0.9 | 20 | 0.3 | 62 | 0.7 |
| *Desulfovibrio* | 0 | 0.0 | 123 | 5.5 | 409 | 5.1 | 1285 | 14.9 |
| *Dysgonomonas* | 0 | 0.0 | 470 | 21.0 | 2558 | 32.0 | 972 | 11.3 |
| *Ferruginibacter* | 149 | 2.9 | 0 | 0.0 | 1 | 0.0 | 0 | 0.0 |
| *Fusibacter* | 304 | 5.9 | 0 | 0.0 | 0 | 0.0 | 0 | 0.0 |
| *Gemmatimonas* | 13 | 0.3 | 0 | 0.0 | 0 | 0.0 | 0 | 0.0 |
| *Geobacter* | 0 | 0.0 | 129 | 5.8 | 0 | 0.0 | 16 | 0.2 |
| Gp4 | 30 | 0.6 | 0 | 0.0 | 0 | 0.0 | 1 | 0.0 |
| *Gracilibacter* | 0 | 0.0 | 9 | 0.4 | 9 | 0.1 | 21 | 0.2 |
| *Haliscomenobacter* | 85 | 1.6 | 0 | 0.0 | 0 | 0.0 | 0 | 0.0 |
| *Hoyosella* | 34 | 0.7 | 1 | 0.0 | 0 | 0.0 | 0 | 0.0 |
| *Hydrogenoanaerobacterium* | 0 | 0.0 | 0 | 0.0 | 2 | 0.0 | 10 | 0.1 |
| *Hydrogenophaga* | 91 | 1.8 | 0 | 0.0 | 4 | 0.1 | 3 | 0.0 |
| *Hyphomicrobium* | 4 | 0.1 | 10 | 0.4 | 20 | 0.3 | 43 | 0.5 |
| *Hyphomonas* | 10 | 0.2 | 1 | 0.0 | 0 | 0.0 | 0 | 0.0 |
| *Legionella* | 28 | 0.5 | 0 | 0.0 | 13 | 0.2 | 5 | 0.1 |
| *Marinomonas* | 640 | 12.4 | 1 | 0.0 | 0 | 0.0 | 0 | 0.0 |
| *Methylobacillus* | 0 | 0.0 | 1 | 0.0 | 5 | 0.1 | 0 | 0.0 |
| *Micropruina* | 0 | 0.0 | 1 | 0.0 | 1 | 0.0 | 25 | 0.3 |
| *Nitrosococcus* | 10 | 0.2 | 0 | 0.0 | 0 | 0.0 | 0 | 0.0 |
| *Nitrospira* | 23 | 0.4 | 0 | 0.0 | 0 | 0.0 | 0 | 0.0 |
| OD1 | 25 | 0.5 | 0 | 0.0 | 0 | 0.0 | 0 | 0.0 |
| *Oscillibacter* | 0 | 0.0 | 7 | 0.3 | 1 | 0.0 | 20 | 0.2 |
| *Owenweeksia* | 54 | 1.0 | 0 | 0.0 | 0 | 0.0 | 0 | 0.0 |
| *Parabacteroides* | 1 | 0.0 | 46 | 2.1 | 82 | 1.0 | 524 | 6.1 |

**Table S1** Continued.

| Taxonomic group | Activated sludge | | Anode biofilm | | Cathode biofilm | | Electrolyte | |
| --- | --- | --- | --- | --- | --- | --- | --- | --- |
|  | No. | % | No. | % | No. | % | No. | % |
| *Paracoccus* | 11 | 0.2 | 4 | 0.2 | 3 | 0.0 | 7 | 0.1 |
| *Petrimonas* | 0 | 0.0 | 7 | 0.3 | 0 | 0.0 | 2 | 0.0 |
| *Pleomorphomonas* | 0 | 0.0 | 11 | 0.5 | 384 | 4.8 | 25 | 0.3 |
| *Proteocatella* | 41 | 0.8 | 0 | 0.0 | 0 | 0.0 | 0 | 0.0 |
| *Pseudomonas* | 277 | 5.3 | 0 | 0.0 | 2 | 0.0 | 0 | 0.0 |
| *Pseudoxanthomonas* | 0 | 0.0 | 10 | 0.4 | 146 | 1.8 | 42 | 0.5 |
| *Rhizobium* | 0 | 0.0 | 2 | 0.1 | 31 | 0.4 | 6 | 0.1 |
| *Sedimentibacter* | 24 | 0.5 | 0 | 0.0 | 0 | 0.0 | 0 | 0.0 |
| *Shewanella* | 135 | 2.6 | 0 | 0.0 | 0 | 0.0 | 0 | 0.0 |
| *Simplicispira* | 16 | 0.3 | 0 | 0.0 | 0 | 0.0 | 0 | 0.0 |
| *Sporacetigenium* | 202 | 3.9 | 8 | 0.4 | 2 | 0.0 | 3 | 0.0 |
| *Sporomusa* | 0 | 0.0 | 370 | 16.5 | 1725 | 21.6 | 1558 | 18.1 |
| *Sporotalea* | 0 | 0.0 | 38 | 1.7 | 6 | 0.1 | 0 | 0.0 |
| *Stenotrophomonas* | 0 | 0.0 | 0 | 0.0 | 12 | 0.2 | 0 | 0.0 |
| *Sulfurimonas* | 117 | 2.3 | 0 | 0.0 | 0 | 0.0 | 0 | 0.0 |
| TM7 | 12 | 0.2 | 0 | 0.0 | 0 | 0.0 | 1 | 0.0 |
| *Terrimonas* | 3 | 0.1 | 1 | 0.0 | 10 | 0.1 | 1 | 0.0 |
| *Thauera* | 63 | 1.2 | 2 | 0.1 | 0 | 0.0 | 1 | 0.0 |
| *Thiobacillus* | 18 | 0.3 | 2 | 0.1 | 0 | 0.0 | 0 | 0.0 |
| *Thiomonas* | 0 | 0.0 | 30 | 1.3 | 19 | 0.2 | 116 | 1.3 |
| *Tissierella* | 23 | 0.4 | 0 | 0.0 | 1 | 0.0 | 1 | 0.0 |
| *Truepera* | 34 | 0.7 | 2 | 0.1 | 0 | 0.0 | 0 | 0.0 |
| *Xanthobacter* | 0 | 0.0 | 8 | 0.4 | 184 | 2.3 | 5 | 0.1 |
| Others | 2406 | 46.4 | 746 | 33.3 | 1890 | 23.6 | 3158 | 36.7 |
| Total | 5181 | 100.0 | 2283 | 100.0 | 7996 | 100.0 | 8610 | 100.0 |
