# Supplementary material for: Metagenomic Analyses Reveal the Involvement of Syntrophic Consortia in Methanol/Electricity Conversion in Microbial Fuel Cells
Source: PLoS One. 2014 May 22;9(5):e98425. doi: 10.1371/journal.pone.0098425 (PMC4031174; doi:10.1371/journal.pone.0098425)
Supplement: Table S2 — (DOCX) [file pone.0098425.s005.docx]

**Table S2** Large contigs (>50 kb) obtained from the anode biofilm in the methanol-fed MFC.

| Contig ID | Length (bp) | MEGAN | |  | BLSOM | BLAST search for genes in contig ^a^ |
| --- | --- | --- | --- | --- | --- | --- |
|  |  | Phylum | Class |  | Phylum |  |
| NODE_10218 | 586573 | *Firmicutes* | *Clostridia* |  | *Firmicutes* | MttB |
| NODE_13136 | 517389 | *Firmicutes* | *Negativicutes* |  | *Firmicutes* | ACK, NaDH |
| NODE_772 | 509211 | *Firmicutes* | *Negativicutes* |  | *Firmicutes* | Cyt bd complex, NaDH, ODH, MDH |
| NODE_1561 | 396697 | *Firmicutes* | *Negativicutes* |  | *Firmicutes* | MDH, CST |
| NODE_3843 | 380872 | *Firmicutes* | *Negativicutes* |  | *Firmicutes* |  |
| NODE_1193 | 303535 | *Firmicutes* | *Negativicutes* |  | *Firmicutes* |  |
| NODE_146 | 294606 | *Proteobacteria* | *Deltaproteobacteria* |  | *Proteobacteria* | NaDH, ODH, PpcA, OmcE, MHC |
| NODE_741 | 288265 | *Proteobacteria* | No hit |  | *Firmicutes* | SIR |
| NODE_12592 | 283961 | *Firmicutes* | *Negativicutes* |  | *Firmicutes* | NaDH, SIR |
| NODE_26787 | 274615 | *Firmicutes* | *Negativicutes* |  | *Firmicutes* | SCS, ODH |
| NODE_144 | 261709 | *Firmicutes* | *Negativicutes* |  | *Firmicutes* |  |
| NODE_6836 | 260423 | *Firmicutes* | *Negativicutes* |  | *Firmicutes* |  |
| NODE_543 | 252753 | *Proteobacteria* | *Alphaproteobacteria* |  | *Proteobacteria* | NaDH, ODH, CST |
| NODE_25 | 252461 | *Chloroflexi* | *Chloroflexi* |  | *Proteobacteria* | NIR |
| NODE_632 | 248183 | *Firmicutes* | *Clostridia* |  | *Firmicutes* | MttB |
| NODE_48727 | 244512 | *Firmicutes* | *Negativicutes* |  | *Firmicutes* | ACK, PAC |
| NODE_47099 | 244390 | *Bacteroidetes* | *Bacteroidia* |  | *Bacteroidetes* |  |
| NODE_3784 | 243446 | *Firmicutes* | *Negativicutes* |  | *Firmicutes* | FUM |
| NODE_348 | 242502 | *Firmicutes* | *Negativicutes* |  | *Firmicutes* | MttB, MttC, MTR, CODH, NIR |
| NODE_56486 | 238405 | *Firmicutes* | *Negativicutes* |  | *Firmicutes* | SCS, MDH |
| NODE_1556 | 233209 | *Firmicutes* | *Clostridia* |  | *Firmicutes* |  |
| NODE_258 | 229783 | *Firmicutes* | *Negativicutes* |  | *Firmicutes* |  |
| NODE_1113 | 225174 | *Firmicutes* | *Negativicutes* |  | *Firmicutes* |  |
| NODE_1633 | 208052 | *Firmicutes* | *Negativicutes* |  | *Firmicutes* |  |
| NODE_115 | 206679 | *Proteobacteria* | *Deltaproteobacteria* |  | *Proteobacteria* | Cyt bd complex, ACO, ODH, PpcA, MHC |
| NODE_73 | 201870 | *Proteobacteria* | No hit |  | *Proteobacteria* |  |
| NODE_6122 | 201190 | *Firmicutes* | Negativicutes |  | *Firmicutes* |  |
| NODE_2564 | 200606 | *Firmicutes* | Negativicutes |  | *Firmicutes* | CODH, SIR, NIR |
| NODE_1889 | 199101 | *Firmicutes* | Negativicutes |  | *Firmicutes* |  |
| NODE_3040 | 198206 | *Firmicutes* | Negativicutes |  | *Firmicutes* |  |
| NODE_12 | 189471 | *Proteobacteria* | *Deltaproteobacteria* |  | *Proteobacteria* | ACK, PAC, NaDH, ODH |
| NODE_40176 | 189259 | *Bacteroidetes* | *Bacteroidia* |  | *Bacteroidetes* | Cyt bd complex |
| NODE_6895 | 188680 | *Firmicutes* | *Negativicutes* |  | *Firmicutes* | SCS |
| NODE_53 | 187432 | *Proteobacteria* | *Deltaproteobacteria* |  | *Proteobacteria* | ACK, NaDH, Omc |
| NODE_114 | 186059 | *Proteobacteria* | *Alphaproteobacteria* |  | *Proteobacteria* | ACK, PAC, MDH, SCS, SDH, ODH |
| NODE_2096 | 185999 | *Firmicutes* | *Clostridia* |  | *Firmicutes* | MttB, MtaB |
| NODE_5971 | 184242 | *Firmicutes* | *Negativicutes* |  | *Firmicutes* |  |
| NODE_62794 | 182122 | *Bacteroidetes* | *Bacteroidia* |  | *Bacteroidetes* |  |
| NODE_14979 | 177985 | *Firmicutes* | *Negativicutes* |  | *Firmicutes* | ACS, CODH |
| NODE_417 | 171558 | *Proteobacteria* | *Betaproteobacteria* |  | *Firmicutes* |  |
| NODE_4322 | 168610 | *Firmicutes* | *Clostridia* |  | *Firmicutes* | MttB |
| NODE_46 | 168291 | *Proteobacteria* | *Deltaproteobacteria* |  | *Proteobacteria* |  |
| NODE_24 | 167753 | *Proteobacteria* | *Deltaproteobacteria* |  | *Proteobacteria* | CODH |
| NODE_24099 | 166389 | *Firmicutes* | *Negativicutes* |  | *Firmicutes* | MDH, ACO, CST |
| NODE_1670 | 166223 | *Firmicutes* | *Negativicutes* |  | *Firmicutes* |  |
| NODE_57661 | 158505 | *Firmicutes* | *Clostridia* |  | *Firmicutes* |  |
| NODE_86166 | 157254 | *Actinobacteria* | *Actinobacteria* |  | *Actinobacteria* |  |
| NODE_69850 | 156588 | *Firmicutes* | *Clostridia* |  | *Bacteroidetes* |  |
| NODE_79062 | 156134 | Tenericutes | *Mollicutes* |  | *Firmicutes* |  |
| NODE_10272 | 156026 | *Firmicutes* | *Clostridia* |  | *Firmicutes* |  |
| NODE_31245 | 155688 | *Firmicutes* | *Negativicutes* |  | *Firmicutes* | IDH |
| NODE_43968 | 154227 | *Firmicutes* | *Negativicutes* |  | *Firmicutes* | NIR |
| NODE_23623 | 150963 | *Firmicutes* | *Clostridia* |  | *Firmicutes* |  |
| NODE_1017 | 150131 | *Firmicutes* | *Negativicutes* |  | *Firmicutes* |  |
| NODE_3187 | 149567 | *Firmicutes* | *Negativicutes* |  | *Firmicutes* |  |
| NODE_78332 | 148517 | *Firmicutes* | *Clostridia* |  | *Firmicutes* |  |
| NODE_22013 | 148361 | *Firmicutes* | *Clostridia* |  | *Firmicutes* |  |
| NODE_207 | 148223 | *Proteobacteria* | *Alphaproteobacteria* |  | *Proteobacteria* | Cyt c oxidase, Cyt bo |
| NODE_15 | 147138 | *Proteobacteria* | *Alphaproteobacteria* |  | *Proteobacteria* |  |
| NODE_381 | 146654 | *Proteobacteria* | *Alphaproteobacteria* |  | *Proteobacteria* |  |
| NODE_155 | 146153 | *Proteobacteria* | *Deltaproteobacteria* |  | *Proteobacteria* |  |
| NODE_34343 | 143856 | *Firmicutes* | *Negativicutes* |  | *Firmicutes* | MtaB, NaDH |

**Table S2** Continued.

| Contig ID | Length (bp) | MEGAN | |  | BLSOM | BLAST search for genes in contig ^a^ |
| --- | --- | --- | --- | --- | --- | --- |
|  |  | Phylum | Class |  | Phylum |  |
| NODE_71891 | 140760 | *Bacteroidetes* | *Bacteroidia* |  | *Bacteroidetes* |  |
| NODE_190 | 137301 | *Bacteroidetes* | *Bacteroidia* |  | *Bacteroidetes* | NIR |
| NODE_311 | 135611 | *Proteobacteria* | *Alphaproteobacteria* |  | *Proteobacteria* |  |
| NODE_149 | 134607 | *Proteobacteria* | *Alphaproteobacteria* |  | *Proteobacteria* | NaDH |
| NNODE_7602 | 134293 | *Firmicutes* | *Negativicutes* |  | *Firmicutes* |  |
| NODE_27528 | 133840 | *Firmicutes* | *Negativicutes* |  | *Firmicutes* |  |
| NODE_135 | 133377 | *Proteobacteria* | *Alphaproteobacteria* |  | *Proteobacteria* | IDH, FUM |
| NODE_1106 | 132924 | *Firmicutes* | *Clostridia* |  | *Firmicutes* | MtaB |
| NODE_2951 | 132222 | *Firmicutes* | *Negativicutes* |  | *Firmicutes* | MDH, CST |
| NODE_84996 | 132206 | *Bacteroidetes* | *Bacteroidia* |  | *Bacteroidetes* |  |
| NODE_40 | 129403 | *Proteobacteria* | *Deltaproteobacteria* |  | *Proteobacteria* |  |
| NODE_87002 | 129305 | *Bacteroidetes* | *Bacteroidia* |  | *Bacteroidetes* |  |
| NODE_68930 | 127175 | *Firmicutes* | *Clostridia* |  | *Firmicutes* |  |
| NODE_69192 | 126184 | *Actinobacteria* | *Actinobacteria* |  | *Actinobacteria* |  |
| NODE_372 | 124345 | *Proteobacteria* | *Alphaproteobacteria* |  | *Proteobacteria* |  |
| NODE_42542 | 123181 | *Firmicutes* | *Clostridia* |  | *Proteobacteria* |  |
| NODE_3223 | 120852 | *Firmicutes* | *Negativicutes* |  | *Firmicutes* |  |
| NODE_208 | 119725 | *Proteobacteria* | *Deltaproteobacteria* |  | *Proteobacteria* | MHC |
| NODE_245 | 118223 | *Proteobacteria* | *Betaproteobacteria* |  | *Proteobacteria* |  |
| NODE_6344 | 117266 | *Proteobacteria* | *Betaproteobacteria* |  | *Proteobacteria* |  |
| NODE_172 | 115284 | *Firmicutes* | *Negativicutes* |  | *Firmicutes* |  |
| NODE_27263 | 112322 | *Firmicutes* | *Negativicutes* |  | *Firmicutes* |  |
| NODE_118 | 110818 | *Proteobacteria* | *Deltaproteobacteria* |  | *Proteobacteria* |  |
| NODE_720 | 110045 | *Proteobacteria* | *Alphaproteobacteria* |  | *Proteobacteria* | NaDH |
| NODE_1290 | 109131 | *Firmicutes* | *Negativicutes* |  | *Firmicutes* | ACK |
| NODE_80524 | 107876 | *Firmicutes* | *Clostridia* |  | *Firmicutes* |  |
| NODE_53989 | 106235 | *Bacteroidetes* | *Bacteroidia* |  | *Bacteroidetes* |  |
| NODE_1959 | 105542 | *Proteobacteria* | *Betaproteobacteria* |  | *Proteobacteria* | IDH, ODH, SDH |
| NODE_4951 | 104731 | *Firmicutes* | *Clostridia* |  | *Firmicutes* |  |
| NODE_2683 | 102438 | *Firmicutes* | No hit |  | *Firmicutes* | CST, SDH |
| NODE_74016 | 101893 | *Bacteroidetes* | *Bacteroidia* |  | *Bacteroidetes* |  |
| NODE_5 | 99845 | *Bacteroidetes* | *Bacteroidia* |  | *Bacteroidetes* |  |
| NODE_24581 | 99643 | *Firmicutes* | *Negativicutes* |  | *Firmicutes* |  |
| NODE_16483 | 99308 | *Firmicutes* | *Negativicutes* |  | *Firmicutes* | SIR |
| NODE_17427 | 96687 | *Actinobacteria* | *Actinobacteria* |  | *Actinobacteria* |  |
| NODE_52620 | 96045 | *Firmicutes* | *Clostridia* |  | *Proteobacteria* |  |
| NODE_51303 | 95695 | *Bacteroidetes* | *Bacteroidia* |  | *Bacteroidetes* |  |
| NODE_74559 | 95105 | *Firmicutes* | *Clostridia* |  | *Firmicutes* |  |
| NODE_224 | 94878 | *Proteobacteria* | *Deltaproteobacteria* |  | *Proteobacteria* |  |
| NODE_74288 | 94687 | *Firmicutes* | *Clostridia* |  | *Proteobacteria* |  |
| NODE_507 | 94366 | *Proteobacteria* | *Alphaproteobacteria* |  | *Proteobacteria* |  |
| NODE_1351 | 93192 | *Firmicutes* | *Negativicutes* |  | *Firmicutes* |  |
| NODE_889 | 92948 | *Proteobacteria* | No hit |  | *Firmicutes* |  |
| NODE_7 | 92675 | *Bacteroidetes* | *Bacteroidia* |  | *Bacteroidetes* |  |
| NODE_69783 | 92381 | *Bacteroidetes* | *Bacteroidia* |  | *Bacteroidetes* |  |
| NODE_1065 | 91901 | *Firmicutes* | No hit |  | *Firmicutes* |  |
| NODE_53563 | 90758 | *Firmicutes* | *Negativicutes* |  | *Firmicutes* |  |
| NODE_54498 | 90684 | *Bacteroidetes* | *Bacteroidia* |  | *Bacteroidetes* |  |
| NODE_68801 | 90206 | *Bacteroidetes* | *Bacteroidia* |  | *Bacteroidetes* |  |
| NODE_733 | 89560 | *Proteobacteria* | *Alphaproteobacteria* |  | *Proteobacteria* | NaDH, ODH |
| NODE_84527 | 89532 | *Bacteroidetes* | *Bacteroidia* |  | *Bacteroidetes* |  |
| NODE_16 | 88874 | *Proteobacteria* | *Deltaproteobacteria* |  | *Proteobacteria* |  |
| NODE_69785 | 87563 | *Actinobacteria* | *Actinobacteria* |  | *Actinobacteria* |  |
| NODE_707 | 87254 | *Proteobacteria* | *Betaproteobacteria* |  | *Proteobacteria* | FUM |
| NODE_633 | 87169 | *Bacteroidetes* | *Bacteroidia* |  | *Bacteroidetes* |  |
| NODE_3785 | 86932 | *Firmicutes* | *Negativicutes* |  | *Firmicutes* |  |
| NODE_83635 | 86804 | *Bacteroidetes* | *Bacteroidia* |  | *Bacteroidetes* |  |
| NODE_157 | 86449 | *Proteobacteria* | *Alphaproteobacteria* |  | *Proteobacteria* | Cyt c oxidase |
| NODE_76114 | 86384 | *Actinobacteria* | *Actinobacteria* |  | *Actinobacteria* |  |
| NODE_36569 | 85992 | *Actinobacteria* | *Actinobacteria* |  | *Actinobacteria* |  |
| NODE_45767 | 85706 | *Bacteroidetes* | *Bacteroidia* |  | *Bacteroidetes* |  |
| NODE_6446 | 85636 | *Firmicutes* | *Clostridia* |  | *Firmicutes* | MttB |

**Table S2** Continued.

| Contig ID | Length (bp) | MEGAN | |  | BLSOM | BLAST search for genes in contig ^a^ |
| --- | --- | --- | --- | --- | --- | --- |
|  |  | Phylum | Class |  | Phylum |  |
| NODE_812 | 85264 | *Proteobacteria* | *Alphaproteobacteria* |  | *Proteobacteria* | Cyt c oxidase ccb3 |
| NODE_41326 | 84770 | *Firmicutes* | *Negativicutes* |  | *Firmicutes* |  |
| NODE_246 | 84650 | *Firmicutes* | *Negativicutes* |  | *Proteobacteria* |  |
| NODE_342 | 84508 | *Proteobacteria* | *Deltaproteobacteria* |  | *Proteobacteria* |  |
| NODE_75 | 83400 | *Proteobacteria* | *Deltaproteobacteria* |  | *Proteobacteria* |  |
| NODE_450 | 83362 | *Proteobacteria* | *Deltaproteobacteria* |  | *Proteobacteria* | NIR |
| NODE_441 | 82081 | *Proteobacteria* | *Deltaproteobacteria* |  | *Proteobacteria* | IDH |
| NODE_79506 | 81901 | *Firmicutes* | No hit |  | *Firmicutes* |  |
| NODE_70406 | 81706 | *Bacteroidetes* | *Bacteroidia* |  | *Bacteroidetes* |  |
| NODE_74594 | 81645 | *Actinobacteria* | *Actinobacteria* |  | *Actinobacteria* |  |
| NODE_57967 | 81574 | *Bacteroidetes* | *Bacteroidia* |  | *Bacteroidetes* | NIR |
| NODE_60085 | 81128 | *Bacteroidetes* | *Bacteroidia* |  | *Bacteroidetes* | NaDH, CST |
| NODE_398 | 80241 | *Proteobacteria* | *Alphaproteobacteria* |  | *Proteobacteria* |  |
| NODE_461 | 79939 | *Proteobacteria* | *Betaproteobacteria* |  | *Proteobacteria* |  |
| NODE_8503 | 79676 | *Firmicutes* | *Negativicutes* |  | *Firmicutes* |  |
| NODE_1444 | 79316 | *Firmicutes* | *Clostridia* |  | *Firmicutes* |  |
| NODE_189 | 79252 | *Proteobacteria* | *Betaproteobacteria* |  | *Proteobacteria* | SDH, CST, ACO, ODH |
| NODE_73396 | 78991 | *Bacteroidetes* | *Bacteroidia* |  | *Bacteroidetes* |  |
| NODE_11333 | 78738 | *Firmicutes* | *Clostridia* |  | *Firmicutes* |  |
| NODE_804 | 78245 | *Firmicutes* | *Negativicutes* |  | *Firmicutes* |  |
| NODE_60380 | 77715 | *Bacteroidetes* | *Bacteroidia* |  | *Bacteroidetes* |  |
| NODE_67070 | 77462 | *Firmicutes* | *Erysipelotrichi* |  | *Firmicutes* |  |
| NODE_1970 | 77398 | *Firmicutes* | *Negativicutes* |  | *Firmicutes* |  |
| NODE_503 | 76803 | *Proteobacteria* | *Alphaproteobacteria* |  | *Proteobacteria* |  |
| NODE_70116 | 76404 | *Actinobacteria* | *Actinobacteria* |  | *Actinobacteria* | Cyt c oxidase ccb3 |
| NODE_44767 | 75719 | *Actinobacteria* | *Actinobacteria* |  | *Actinobacteria* | CST |
| NODE_6840 | 75398 | *Firmicutes* | *Negativicutes* |  | *Firmicutes* |  |
| NODE_85035 | 75166 | *Bacteroidetes* | *Bacteroidia* |  | *Bacteroidetes* |  |
| NODE_53473 | 75089 | *Bacteroidetes* | *Bacteroidia* |  | *Bacteroidetes* |  |
| NODE_892 | 74718 | *Firmicutes* | *Clostridia* |  | *Firmicutes* |  |
| NODE_7138 | 74627 | *Firmicutes* | *Negativicutes* |  | *Firmicutes* |  |
| NODE_4308 | 74546 | *Bacteroidetes* | *Bacteroidia* |  | *Bacteroidetes* |  |
| NODE_177 | 74403 | *Bacteroidetes* | *Bacteroidia* |  | *Bacteroidetes* |  |
| NODE_8 | 74226 | *Firmicutes* | *Negativicutes* |  | *Firmicutes* |  |
| NODE_65651 | 74088 | *Actinobacteria* | *Actinobacteria* |  | *Actinobacteria* |  |
| NODE_131 | 73637 | *Proteobacteria* | *Betaproteobacteria* |  | *Proteobacteria* | MTR |
| NODE_63727 | 73488 | *Firmicutes* | *Clostridia* |  | *Firmicutes* |  |
| NODE_13588 | 72171 | *Firmicutes* | *Negativicutes* |  | *Firmicutes* |  |
| NODE_455 | 72154 | *Proteobacteria* | *Betaproteobacteria* |  | *Proteobacteria* |  |
| NODE_622 | 72143 | *Firmicutes* | *Clostridia* |  | *Firmicutes* |  |
| NODE_264 | 71978 | *Proteobacteria* | *Betaproteobacteria* |  | *Proteobacteria* |  |
| NODE_9557 | 71809 | *Firmicutes* | *Negativicutes* |  | *Firmicutes* | SCS |
| NODE_15646 | 71588 | *Firmicutes* | *Negativicutes* |  | *Firmicutes* |  |
| NODE_48750 | 71314 | *Firmicutes* | *Clostridia* |  | *Firmicutes* |  |
| NODE_14424 | 71146 | *Firmicutes* | *Clostridia* |  | *Firmicutes* | ACS |
| NODE_186 | 70967 | *Firmicutes* | *Negativicutes* |  | *Firmicutes* |  |
| NODE_894 | 70218 | *Proteobacteria* | *Betaproteobacteria* |  | *Proteobacteria* |  |
| NODE_193 | 69476 | *Firmicutes* | *Negativicutes* |  | *Firmicutes* | NaDH |
| NODE_329 | 68819 | *Proteobacteria* | *Deltaproteobacteria* |  | *Proteobacteria* | Cyt c oxidase |
| NODE_11712 | 68811 | *Firmicutes* | *Negativicutes* |  | *Firmicutes* |  |
| NODE_51831 | 68708 | *Actinobacteria* | *Actinobacteria* |  | *Actinobacteria* |  |
| NODE_334 | 68603 | *Proteobacteria* | *Alphaproteobacteria* |  | *Proteobacteria* |  |
| NODE_393 | 68265 | *Bacteroidetes* | *Bacteroidia* |  | *Bacteroidetes* |  |
| NODE_103 | 68206 | *Proteobacteria* | *Deltaproteobacteria* |  | *Proteobacteria* | Cyt c oxidase |
| NODE_46678 | 68095 | *Actinobacteria* | *Actinobacteria* |  | *Actinobacteria* |  |
| NODE_9102 | 68066 | *Firmicutes* | *Negativicutes* |  | *Firmicutes* |  |
| NODE_8301 | 68064 | *Bacteroidetes* | *Bacteroidia* |  | *Bacteroidetes* |  |
| NODE_82817 | 67614 | No hit | No hit |  | *Firmicutes* |  |
| NODE_60632 | 67591 | *Actinobacteria* | *Actinobacteria* |  | *Actinobacteria* |  |
| NODE_58708 | 67370 | *Firmicutes* | *Clostridia* |  | *Firmicutes* |  |
| NODE_214 | 67082 | *Proteobacteria* | *Alphaproteobacteria* |  | *Proteobacteria* |  |
| NODE_853 | 67046 | No hit | *No hit* |  | *Bacteroidetes* |  |

**Table S2** Continued.

| Contig ID | Length (bp) | MEGAN | |  | BLSOM | BLAST search for genes in contig ^a^ |
| --- | --- | --- | --- | --- | --- | --- |
|  |  | Phylum | Class |  | Phylum |  |
| NODE_2817 | 66766 | *Proteobacteria* | *Betaproteobacteria* |  | *Firmicutes* |  |
| NODE_46909 | 66360 | *Bacteroidetes* | *Bacteroidia* |  | *Bacteroidetes* |  |
| NODE_88 | 66189 | *Proteobacteria* | *Deltaproteobacteria* |  | *Proteobacteria* |  |
| NODE_626 | 66113 | *Proteobacteria* | *Deltaproteobacteria* |  | *Proteobacteria* |  |
| NODE_80917 | 65995 | No hit | No hit |  | *Firmicutes* |  |
| NODE_449 | 65977 | *Proteobacteria* | *Betaproteobacteria* |  | *Proteobacteria* | Cyt c oxidase |
| NODE_61063 | 65862 | *Bacteroidetes* | *Bacteroidia* |  | *Bacteroidetes* |  |
| NODE_8884 | 65772 | *Firmicutes* | *Clostridia* |  | *Firmicutes* | MttB |
| NODE_54476 | 65719 | *Actinobacteria* | *Actinobacteria* |  | *Actinobacteria* |  |
| NODE_306 | 65575 | *Proteobacteria* | *Deltaproteobacteria* |  | *Proteobacteria* | OmcE, MHC |
| NODE_11803 | 65511 | *Proteobacteria* | *Betaproteobacteria* |  | *Proteobacteria* |  |
| NODE_59450 | 65486 | No hit | No hit |  | *Firmicutes* |  |
| NODE_524 | 65420 | *Bacteroidetes* | *Bacteroidia* |  | *Bacteroidetes* |  |
| NODE_81936 | 65331 | *Actinobacteria* | *Actinobacteria* |  | *Actinobacteria* |  |
| NODE_52918 | 65011 | *Bacteroidetes* | *Bacteroidia* |  | *Bacteroidetes* | NaDH |
| NODE_51549 | 64747 | *Firmicutes* | *Clostridia* |  | *Firmicutes* | IDH |
| NODE_46829 | 64452 | *Firmicutes* | *Negativicutes* |  | *Firmicutes* |  |
| NODE_1767 | 63901 | *Proteobacteria* | *Deltaproteobacteria* |  | *Proteobacteria* |  |
| NODE_3261 | 63322 | *Proteobacteria* | *Deltaproteobacteria* |  | *Proteobacteria* |  |
| NODE_260 | 63139 | *Proteobacteria* | *Deltaproteobacteria* |  | *Proteobacteria* | NaDH, CST |
| NODE_72287 | 62915 | *Bacteroidetes* | *Bacteroidia* |  | *Bacteroidetes* |  |
| NODE_14066 | 62881 | *Bacteroidetes* | *Bacteroidia* |  | *Bacteroidetes* |  |
| NODE_759 | 62836 | *Proteobacteria* | *Deltaproteobacteria* |  | *Proteobacteria* |  |
| NODE_176 | 62743 | *Bacteroidetes* | *Bacteroidia* |  | *Bacteroidetes* |  |
| NODE_2423 | 62734 | *Firmicutes* | *Negativicutes* |  | *Firmicutes* |  |
| NODE_52707 | 62624 | *Actinobacteria* | *Actinobacteria* |  | *Actinobacteria* |  |
| NODE_24835 | 62574 | Spirochaetes | *Spirochaetia* |  | *Firmicutes* |  |
| NODE_2604 | 61934 | *Firmicutes* | *Negativicutes* |  | *Firmicutes* |  |
| NODE_77185 | 61327 | *Bacteroidetes* | *Bacteroidia* |  | *Bacteroidetes* |  |
| NODE_47580 | 61190 | *Firmicutes* | *Clostridia* |  | *Firmicutes* |  |
| NODE_84067 | 61164 | *Firmicutes* | *No hit* |  | *Firmicutes* |  |
| NODE_2730 | 61042 | *Proteobacteria* | *Betaproteobacteria* |  | *Proteobacteria* |  |
| NODE_19623 | 60530 | *Proteobacteria* | *Deltaproteobacteria* |  | *Proteobacteria* |  |
| NODE_92 | 59856 | *Proteobacteria* | *Alphaproteobacteria* |  | *Proteobacteria* |  |
| NODE_9819 | 59724 | *Firmicutes* | *Negativicutes* |  | *Firmicutes* | IDH |
| NODE_5732 | 59644 | *Firmicutes* | *Negativicutes* |  | *Firmicutes* |  |
| NODE_2001 | 59348 | *Proteobacteria* | *Betaproteobacteria* |  | *Proteobacteria* |  |
| NODE_66753 | 59244 | *Firmicutes* | *Clostridia* |  | *Proteobacteria* |  |
| NODE_20265 | 59183 | *Actinobacteria* | *Actinobacteria* |  | *Actinobacteria* |  |
| NODE_64973 | 59089 | *Firmicutes* | *Clostridia* |  | *Firmicutes* |  |
| NODE_6778 | 58391 | *Firmicutes* | No hit |  | *Proteobacteria* |  |
| NODE_2978 | 58116 | *Bacteroidetes* | *Bacteroidia* |  | *Bacteroidetes* | ACS |
| NODE_70979 | 57891 | *Firmicutes* | *Clostridia* |  | *Firmicutes* |  |
| NODE_1350 | 57710 | *Proteobacteria* | *Betaproteobacteria* |  | *Proteobacteria* |  |
| NODE_66910 | 57609 | *Proteobacteria* | *Deltaproteobacteria* |  | *Proteobacteria* |  |
| NODE_35706 | 56951 | *Bacteroidetes* | *Bacteroidia* |  | *Bacteroidetes* |  |
| NODE_52057 | 56864 | *Bacteroidetes* | *Bacteroidia* |  | *Bacteroidetes* |  |
| NODE_74137 | 56728 | *Actinobacteria* | *Actinobacteria* |  | *Actinobacteria* |  |
| NODE_237 | 56573 | *Firmicutes* | *Clostridia* |  | *Firmicutes* |  |
| NODE_100 | 56242 | *Bacteroidetes* | *Bacteroidia* |  | *Bacteroidetes* |  |
| NODE_54785 | 56235 | *Actinobacteria* | *Actinobacteria* |  | *Firmicutes* |  |
| NODE_12416 | 56189 | *Firmicutes* | *Negativicutes* |  | *Firmicutes* |  |
| NODE_499 | 56177 | *Bacteroidetes* | *Bacteroidia* |  | *Bacteroidetes* |  |
| NODE_61273 | 56049 | *Firmicutes* | *Bacilli* |  | *Firmicutes* |  |
| NODE_3938 | 55966 | *Firmicutes* | *Negativicutes* |  | *Firmicutes* |  |
| NODE_91 | 55841 | No hit | No hit |  | *Proteobacteria* |  |
| NODE_60766 | 55827 | *Actinobacteria* | *Actinobacteria* |  | *Actinobacteria* |  |
| NODE_2412 | 55499 | *Firmicutes* | *Negativicutes* |  | *Firmicutes* |  |
| NODE_551 | 55190 | *Proteobacteria* | *Betaproteobacteria* |  | *Proteobacteria* |  |
| NODE_1624 | 55173 | *Firmicutes* | No hit |  | *Bacteroidetes* |  |

**Table S2** Continued.

| Contig ID | Length (bp) | MEGAN | |  | BLSOM | BLAST search for genes in contig ^a^ |
| --- | --- | --- | --- | --- | --- | --- |
|  |  | Phylum | Class |  | Phylum |  |
| NODE_77979 | 55161 | *Firmicutes* | No hit |  | *Firmicutes* |  |
| NODE_5521 | 55030 | *Firmicutes* | *Negativicutes* |  | *Firmicutes* | MDH |
| NODE_5810 | 54126 | *Bacteroidetes* | *Bacteroidia* |  | *Bacteroidetes* |  |
| NODE_607 | 53874 | *Proteobacteria* | *Betaproteobacteria* |  | *Proteobacteria* |  |
| NODE_81 | 53689 | *Proteobacteria* | *Deltaproteobacteria* |  | *Proteobacteria* | SIR |
| NODE_75060 | 53477 | *Bacteroidetes* | *Bacteroidia* |  | *Bacteroidetes* |  |
| NODE_3 | 53384 | *Bacteroidetes* | No hit |  | *Bacteroidetes* |  |
| NODE_77439 | 53340 | No hit | No hit |  | *Proteobacteria* |  |
| NODE_123 | 53193 | *Proteobacteria* | *Alphaproteobacteria* |  | *Proteobacteria* |  |
| NODE_8316 | 52442 | No hit | No hit |  | *Firmicutes* |  |
| NODE_79467 | 52398 | *Firmicutes* | *Clostridia* |  | *Firmicutes* |  |
| NODE_827 | 52314 | *Proteobacteria* | *Betaproteobacteria* |  | *Proteobacteria* |  |
| NODE_122 | 52283 | *Proteobacteria* | *Deltaproteobacteria* |  | *Proteobacteria* | ACK, NaDH, SDH |
| NODE_65431 | 52197 | *Bacteroidetes* | *Bacteroidia* |  | *Bacteroidetes* |  |
| NODE_36059 | 52070 | *Bacteroidetes* | *Bacteroidia* |  | *Bacteroidetes* |  |
| NODE_21064 | 51697 | *Firmicutes* | *Clostridia* |  | *Firmicutes* |  |
| NODE_16045 | 51601 | *Firmicutes* | *Negativicutes* |  | *Firmicutes* |  |
| NODE_2769 | 51312 | *Proteobacteria* | *Deltaproteobacteria* |  | *Proteobacteria* | CODH |
| NODE_72 | 51137 | *Proteobacteria* | *Betaproteobacteria* |  | *Proteobacteria* |  |
| NODE_44 | 51108 | *Proteobacteria* | *Deltaproteobacteria* |  | *Proteobacteria* |  |
| NODE_35368 | 50966 | *Bacteroidetes* | *Bacteroidia* |  | *Bacteroidetes* |  |
| NODE_1028 | 50862 | *Proteobacteria* | *Alphaproteobacteria* |  | *Proteobacteria* | SIR |
| NODE_292 | 50487 | *Proteobacteria* | *Alphaproteobacteria* |  | *Proteobacteria* |  |
| NODE_19 | 50486 | *Proteobacteria* | *Deltaproteobacteria* |  | *Proteobacteria* |  |
| NODE_21435 | 50394 | *Firmicutes* | No hit |  | *Proteobacteria* |  |
| NODE_1599 | 50297 | *Proteobacteria* | *Betaproteobacteria* |  | *Proteobacteria* |  |
| NODE_1312 | 50279 | *Proteobacteria* | *Betaproteobacteria* |  | *Proteobacteria* | SCS |
| NODE_231 | 50230 | *Proteobacteria* | *Betaproteobacteria* |  | *Proteobacteria* |  |
| NODE_78977 | 50230 | *Bacteroidetes* | *Bacteroidia* |  | *Bacteroidetes* |  |
| NODE_344 | 50212 | *Proteobacteria* | *Deltaproteobacteria* |  | *Proteobacteria* | ACO, ODH |
| NODE_43 | 50188 | *Bacteroidetes* | *Bacteroidia* |  | *Proteobacteria* |  |
| NODE_78867 | 50097 | *Actinobacteria* | *Actinobacteria* |  | *Actinobacteria* |  |

^a^ Abbreviations are as follows: NaDH, NADH dehydrogenase; ODH, 2-oxoglutarate dehydrogenase; IDH, isocitrate dehydrogenase; ACO, aconitase; MDH, malate dehydrogenase; FUM, fumarase; SDH, succinate dehydrogenase; SCS, succinyl-CoA synthase; CIS, citrate synthase; ACK, acetate kinase; PAC, phosphate acetyltransferase; ACS, acetyl-CoA synthase; MTR, methylenetetrahydrofolate reductase; CODH, carbon-monoxide; cyt, cytochrome; Ppc, periplasmic cyt c; Omc, outer-membrane cyt c; MHC, multiheme cytochrome; Mtt, tetramethylammonium methyltransferase; Mta, methanol methyltransferase; SIR, sulfite reductase; NIR, nitrate reductase.
